# Unprecedented confinement time of electron plasmas with a purely toroidal magnetic field in SMARTEX-C

**DOI:** 10.1038/s41598-023-44849-2

**Published:** 2023-11-03

**Authors:** Lavkesh Lachhvani, Sambaran Pahari, Rajiv Goswami, Yogesh G. Yeole, Minsha Shah, Nikhil Mohurle, Prabal K. Chattopadhyay

**Affiliations:** 1 Institute for Plasma Research, A CI of Homi Bhabha National Institute, Bhat, Gandhinagar, Gujarat 382428 India; 2 Bhabha Atomic Research Centre, HBNI, Visakhapatnam, 530012 India

**Keywords:** Physics, Plasma physics, Magnetically confined plasmas

## Abstract

Confinement time of electron plasmas trapped using a purely toroidal magnetic field has been found to exceed ${100}\, \hbox {s}$ in a small aspect ratio ($R_{o}/a \sim {1.59}$, $R_o$ and *a* are device major and minor radius, respectively), partial torus. It improves upon the previously reported confinement time by nearly two orders of magnitude. Lifetime is estimated from the frequency scaling of the linear diocotron mode launched from sections of the wall, that are also used for mode diagnostics. Confinement improves as neutral pressures are reduced to $< 5 \times 10^{-10} \hbox{mbar}$ in the presence of a steady state magnetic field of 200 Gauss ($\sim {60}\, \hbox {s}$ with droop $< 0.1\%$) at ${100}\, \hbox {V}$ electron injection energies. With reduced pressures the role of (ion driven) instability diminishes and loss mechanisms resulting from elastic electron–neutral (e–n) and the ubiquitous electron–electron (e–e) scattering seem to play an important role which suggests low electron temperatures. The contribution to electron population resulting from the ionization of background neutral gas at these temperatures and pressures are expected to be insignificant and is corroborated in our experiments.

## Introduction

The excellent confinement of electron plasmas in cylindrical traps had unleashed a plethora of laboratory investigations into their rich collective dynamics in the latter half of previous century^1–4^. This impacted a large number of fundamental studies relevant to diverse fields ranging from atomic physics to (incompressible) fluid dynamics. In contrast, the behaviour and applications of such single species plasmas confined in other geometries and magnetic field topologies remained rather unexplored due to their unknown confinement properties. Historically though, experiments in toroidal electron plasmas, preceded cylindrical plasmas and several applications had also been proposed^5–9^. Besides these, confinement of non-neutral plasma in toroidal geometry and investigating the effects of arbitrary degree of non-neutrality under controlled conditions^10^ was also expected to aid the understanding of transport in neutral plasmas which are of profound interest to the fusion community. In recent times, much of the motivation and interest in toroidal traps seem to follow from the possibility of creating electron-positron pair plasmas^11,12^ due to the expected lack of instabilities in such plasmas and in view of their relevance to astrophysical objects. In addition to this, just like cylindrical electron plasmas in homogeneous magnetic field have served as excellent test-beds for carrying out incompressible fluid dynamics experiments^13,14^, toroidal electron plasma in the presence of an inhomogeneous magnetic field may mimic compressible fluids and has remained an attractive proposition for some time^15^ that certainly merits further investigation. Very recently, feasibility study has been carried out to utilize the trapped electrons for quantum computing and successful trapping of electrons at room temperature in microwave Paul trap is proposed as plausible candidate for the same^16^.

The minimum underlying requirement for achieving any of the stated objectives with toroidal non-neutral plasmas, is a long time confinement. Theoretically, in the presence of a purely toroidal B field, such plasmas are supposed to be in stable equilibrium^17,18^. However, unlike cylindrical plasmas in uniform magnetic field which are governed by robust confinement theorem^19^, plasmas trapped with a purely toroidal B field are thought to be fundamentally limited in their confinement properties due to Magnetic Pumping Transport(MPT). Proposed by O’Neil and Crooks^20^, this radial transport arises due to $E \times B$ drifts of the plasma in a spatially inhomogeneous toroidal B field. In recent times though, computer simulations have constructed a quiescent quasi steady state through entropy maximization which remains close to an absolute equilibrium and stays confined for a long time^21^ in complete as well as partial tori^22^.

Experimentally, a few early initiatives in toroidal traps reported successful trapping to varying degree and demonstrated steady state confinement of a few hundred microseconds,^23–26^ overcoming the single particle drifts. Renewed interest in toroidal traps in the late 90’s and early 2000s, led to a major turnaround. Two of the new traps with purely toroidal magnetic field, were converted into partial torus in order to combine the technique and advantages of cylindrical traps with toroidal geometry. Among them were LNT-I^27^ which was a large aspect ratio device and SMARTEX-C^28^, a small aspect ratio trap. While significant improvement in trapping ranging from few to tens of ms were achieved, yet, till early 2006, the best confinement times reported remained orders of magnitude less than those predicted by MPT theory. Finally, in 2009, with improved operating scenarios like enhanced vacuum, higher magnetic field and a higher degree of symmetry LNT-II successfully confined toroidal electron plasma in a steady state for ${3}\, \hbox {s}$ at a base vacuum of ${1 \times 10^{-8}} \hbox {mbar}$^29–31^. They argued that the confinement time approached the limit set by MPT (for the trap major radius of ${17.4}\, \hbox {cm}$ and assumed temperature of ${1}\, \hbox {eV}$), although no direct evidence of MPT was observed. Results from SMARTEX-C followed, whose reported confinement time (${2.14} \pm {0.1}\, \hbox {s}$) was also very close to their theoretically predicted time scales^32^. Its worth mentioning here that no such theoretical limitations are known to apply to plasmas trapped in other B field topologies like in stellarators or, say, in a torus with levitated dipole magnetic field. Attempts to confine such plasmas on nested flux surfaces in a stellarator^33^ though has been limited to $\sim$ ${90}\, \hbox {ms}$ at ${1.3\,\times \,10^{-9}}\,\hbox {mbar}$ due to ion dynamics. Experiments with a levitated dipole^34^ magnet have however extended the confinement to $\sim {300}\, \hbox {s}$ albeit at slightly higher pressures of ${7\,\times \,10^{-9}}\, \hbox {mbar}$. It may thus appear that low temperature electron plasmas trapped *with purely toroidal B field* are fundamentally constrained and will perhaps never be able to achieve the goals of long time confinement and thermal equilibrium like their cylindrical counterparts and/or other contemporary traps with alternate B field topologies.

This report details an improvement over previously reported results^32^ in the confinement of pure electron plasma by nearly two orders of magnitude in an upgraded SMARTEX-C, where plasma remains in a steady state equilibrium for $\sim {144} \pm {5}\, \hbox {s}$ at 200 Gauss. This is the highest reported confinement time from a trap with purely toroidal B field.

## Experimental set-up

**Figure 1:**
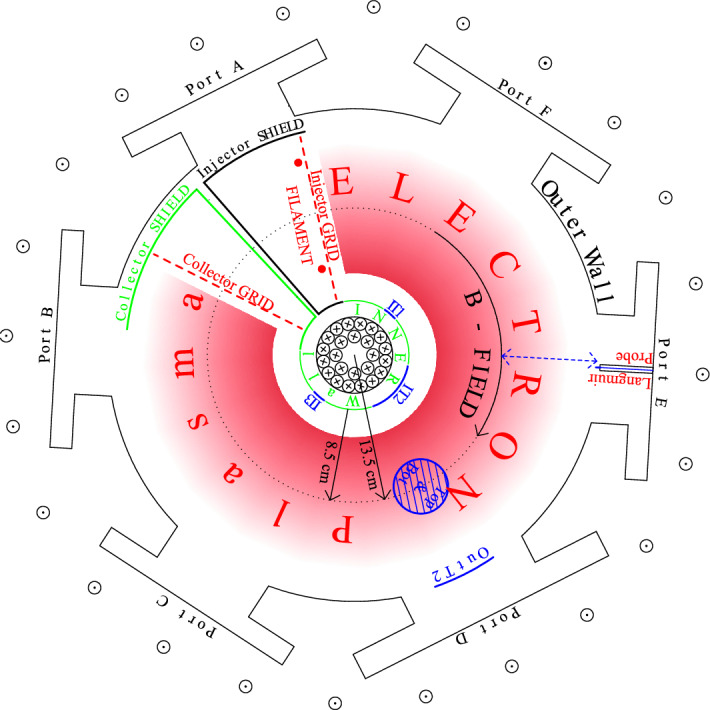
Schematic diagram of SMARTEX-C device (top view).

SMARTEX-C is a partial (C-shaped) toroidal trap with aspect ratio of $R_{0}/{a} \sim 1.59$, trapping electron plasma in an angular arc of $\Phi \sim 315^{\circ }$. The electrode arrangement as shown in Fig.  1 allows us to operate the trap in a “inject-hold-dump” cycle^28^, as is typically carried out in cylindrical Penning-Malmberg traps. The electrons emitted thermionically are injected for a brief period ($\sim {60}\, {\mu \hbox {s}}$) into the arc by turning the injector grid bias positive with respect to the (tungsten) filament. As the grid turns negative, the injection stops. The electrons were injected at $\sim {100}\, \hbox {eV}$ parallel energy and held between negatively biased end-electrodes and radially confined with a purely toroidal B field. A sufficient number of these electrons gives them a collective $E\times B$ drift that helps to overcome the single particle drifts, thus forming a plasma. A nearly steady state B field for $\sim {60}\, \hbox {s}$ could be generated at 100–200 Gauss with droop $< {0.1}\%$. A background pressure of $\sim {4.0\,\times \,10^{-10}}\, \hbox {mbar}$ in the trapping region was maintained with the aid of a Turbo-Molecular Pump (TMP), Cryopump and two Non-Evaporable Getter (NEG) pumps with controlled baking system. It may be noted that presence of neutrals contaminates a pure electron plasma in a number of ways. Neutrals can lead to loss of confinement due to scattering. Ionisation of neutrals can destabilise the diocotron mode and also cause transport. Ionisation may also add to the electron population and misrepresent the estimation of confinement time.

Capacitive probes^35^ (as shown in Fig. 1, for example, IT1, IT2, OutT2, etc) which are essentially parts of the wall, but are insulated from the rest of the wall, are located at various toroidal and poloidal locations. These wall probes are utilized to monitor image currents that can be interpreted to obtain information about any electrostatic activity in the plasma^36^. Additionally, during the quiescent period, these probes have been used to excite normal modes, namely the diocotron modes that are ubiquitously present in such single species plasma. Under linear approximations, the frequency of $m=1$ mode, which represents the azimuthal rotation of a displaced charge cloud, is a good estimate of the charge content.

In cylindrical machines, such modes have been therefore used as a non-destructive diagnostic to estimate the total stored charge. In toroidal machines^23^, if the mode is linear, the total charge content *Q* can be obtained from mode frequency ($m=1$) by using $f_{D} \approx Q/(8\pi ^{3} \varepsilon _{0} R_{0} r^{2} B)$, where *Q* is total stored charge, $-$ *r* is the plasma radius, $\varepsilon _{0}$ is free space permittivity and B is toroidal magnetic field. Note that in toroidal electron plasmas, shift in equilibrium position, if any, has to be accounted for and B field at equilibrium position has to be used. However estimation of lifetime from the time evolution of frequencies is unaffected by this shift as it only scales the exponential function by a factor. The frequency evolution has therefore been used to provide us with an estimation of confinement time^32^.

## Experimental observation and analysis

**Figure 2:**
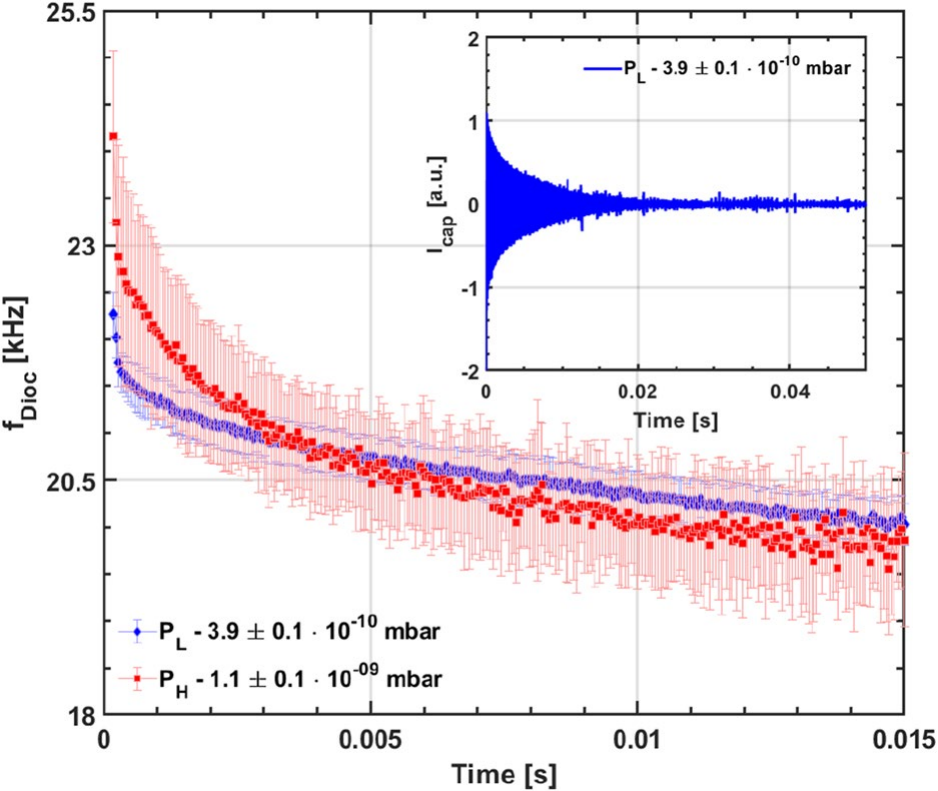
Temporal evolution of initial diocotron mode frequency for two pressures with errorbars as light shades. Inset figure shows the damping of the diocotron instability soon after injection.

In this paper, all the experiments were carried out at fixed values of toroidal magnetic field *B* of 200 G with electron injection at ${100}\, \hbox {V}$, and two different background pressure values of $P_L = {3.9} \pm {0.1\,\times 10^{-10}}\, \hbox {mbar}$ and $P_H = {1.1} \pm {0.1\,\times 10^{-9}}\, \hbox {mbar}$, respectively. In both these cases, a naturally occurring diocotron mode appears almost immediately after the injection of electrons, and whose frequency time evolution is shown in Fig. 2. It is seen that the initial frequencies peak at $\sim {24.1} \pm {0.9}\, \hbox {kHz}$ and $\sim {22.3} \pm {0.3}\, \hbox {kHz}$ for $P_{H}$ and $P_{L}$ respectively. Occurrence of the instability could possibly be due to the electron impact ionization of background neutrals adding some additional electrons to the injected population. This is likely because during and after injection period, the average electron energies are high enough to cause ionization due to the still evolving electron space-charge distribution and its incomplete shielding from the applied end-electrode potentials. During this brief period, an instability attributable to the birth of ions due to the ionization process is seen to arise. It leads to a rapid charge loss and hence we observe the fast decline in frequency as well as amplitude. At high pressures and injected energies, this observed instability due to birth of ions was characterised and delineated from resistive wall instability. It was identified as transient ion resonance instability and was suppressed by controlling the birth of ions in our device^32^. In the meantime, the electron-electron collisions try to establish a thermal equilibrium, independently along and across the field lines, and simultaneously the electrons also lose their energies through inelastic collisions (excitation, dissociation, and ionization) with the neutrals as well as in traversing the sheath that develops at the end-electrodes and is usually more negative than the bulk plasma. As the average electron energy drops well below the ionization threshold of the background gas (hydrogen in our device based on Residual Gas Analyzer data), the role of ionization diminishes; the ion-driven instability is seen to quickly damp. The plasma is then found to approach a quiescent state whose time evolution is described below.

**Figure 3:**
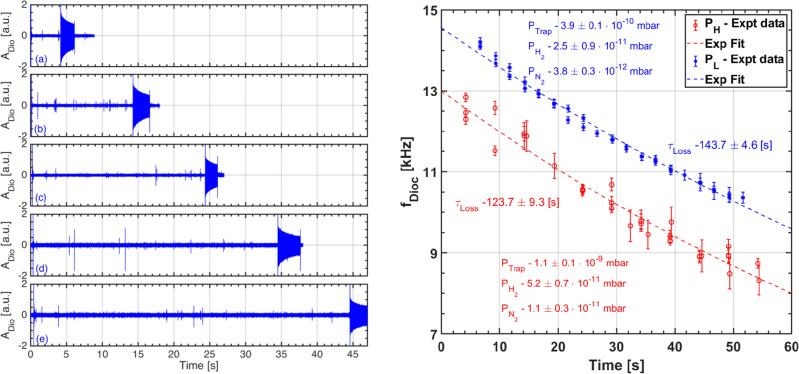
(Left) Time trace of capacitive probe oscillations launching diocotron waves at (**a**) ${4.1}\, \hbox {s}$, (**b**) ${14.1}\, \hbox {s}$, (**c**) ${24.1}\, \hbox {s}$, (**d**) ${34.1}\, \hbox {s}$, and (**e**) ${44.1}\, \hbox {s}$. The plasma is seen to abruptly disappear in (**a**–**e**) after launch because the plasma is dumped deliberately soon after diocotron wave launch. (Right) Observed diocotron mode frequency on capacitive probe along with exponential fit for different pressures at 200 Gauss B-field and injection energy $V_{Inj} = {100}\,\hbox {V}$.

To ascertain the long-term evolution of the pure electron plasma in the quiescent state, diocotron mode was excited at different intervals over 100 shots. Time trace of diocotron mode oscillations excited externally at different time instances has been shown in Fig.  3 (left)a–e. The linear oscillation frequency of the excited diocotron mode is the natural frequency of the electron plasma at that time instance. The frequency evolution reconstructed from these plasma shots for the two pressure values is plotted in Fig.  3 (right). It is observed that the frequencies decrease monotonically for both the cases, but are always lower for $P_{H}$. The confinement times are then obtained by finding the slope of the linear fit applied on the logarithm of the frequency evolution, and are found to be ${143.7} \pm {4.6}\,\hbox {s}$ and ${123.7} \pm {9.3}\,\hbox {s}$ for $P_{L}$ and $P_{H}$, respectively. Note that we do not observe any perceptible instability nor higher frequencies, even for higher pressure values. We therefore surmise that the electron temperature continues to remain low. Thus, unlike the nascent stage, there appears to be no significant ionization of background neutrals through the rest of the trapped duration, which could have led to a contamination of the reported confinement times.

An estimate of temperature range at this stage has been attempted using both direct and indirect methods. A simple particle balance model has been used to obtain an indirect estimate of $T_e$. It describes the time evolution of electron density $n_e$ as $dn_e/dt = n_e \nu _n - n_e/\tau$, where $\nu _n = n_n\langle \sigma v\rangle _i$ is the electron–neutral (e–n) ionization frequency, $n_n$ is the neutral density, $\langle \sigma v\rangle _i$ is the rate coefficient for electron impact ionization of neutrals and is a non-linear function of electron temperature, and $\tau$ is the transport time scale.

**Figure 4:**
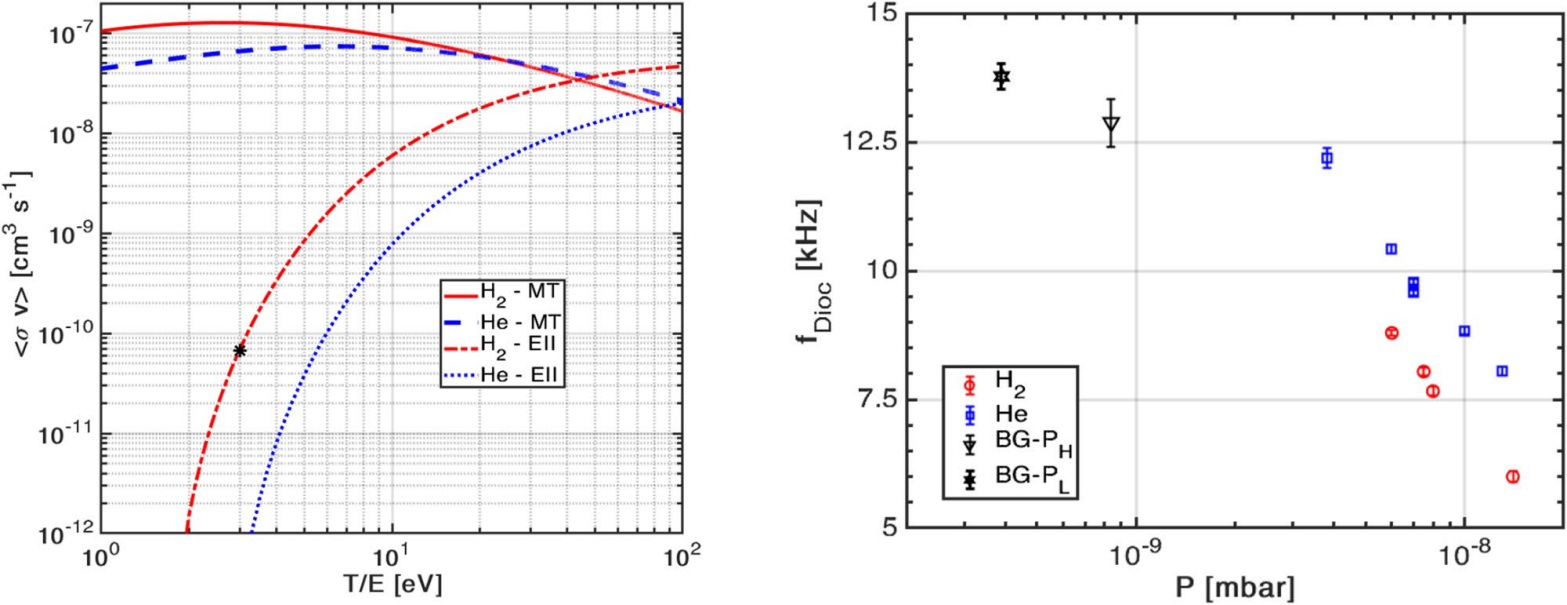
(Left) Rate coefficients for total momentum transfer (MT) electron–neutral scattering and electron impact ionization (EII) for hydrogen and helium. (Right) Launched diocotron mode frequency vs total pressure for hydrogen and helium.

Assuming $n_n$ (for given pressure), $T_e$, and therefore $\nu _n$ to be constant i.e., time-independent, possibly justified during the long and dominant quiescent phase, and using the fact that the experimental confinement time has been estimated from the 1/*e* fall of the density $n_e$, it can be readily shown that $t = \tau /\left( 1 - \nu _n\tau \right)$. Here it must be noted that a more accurate but complex calculation (to be done) would entail solving a Poisson equation with coupled time-dependent particle and energy balance models. We now replace time *t* in the above equation with the respective confinement times obtained from the experimental data, shown in Fig.  3 (right). Furthermore, if we also assume that the underlying major transport mechanism and plasma temperatures remain similar in both the (pressure) cases, then $\tau$ can be equated out and we get a rough estimate of the ionization rate coefficient as $\langle \sigma v\rangle _i \approx \left( 1/t_L - 1/t_H \right) /\left( n_n^{H} - n_n^{L}\right)$, with $t_{L(H)}$ and $n_n^{L(H)}$ being the experimentally known confinement times and neutral densities corresponding to the low and high pressure regimes, respectively. Plugging in the respective values we get $\langle \sigma v \rangle _i \simeq {6.6\,\times 10^{-11}}\,\hbox {cm}^{3}\,\hbox {s}^{-1}$. Now to obtain the electron temperature corresponding to this value, we calculated the variation of a Maxwellian averaged Electron Impact Ionization (EII) rate coefficient with $T_e$ for the (dominant) hydrogen gas molecules $\hbox {H}_{2}$ using a well known cross section database AMJUEL^37^, as shown in Fig.  4 (left). It is found from this plot that $T_e \lesssim {2}\hbox {eV}$ (shown as a black asterisk symbol on the red/dash-dot line).

We have carried out another cross-verification of these results by measuring the peak plasma potential in the trap using a High Impedance (${1}\hbox {G}\Omega$) Langmuir Probe (HILP). It has been used to measure the radial potential profile at a toroidal location of $105^{\circ }$ from the injector grid on the midplane of SMARTEX-C. Measurement of plasma potential using HILP is valid when $n_e \ll n_B$, where $n_B$ is the Brillouin limit on number density ($\sim {2\,\times \,10^{9}}\,\hbox {cm}^{-3}$ for SMARTEX-C parameters). Here, HILP is utilized to measure plasma potential only during or immediately after the injection phase, due to its limitations of a high RC time constant as well as its deleterious effects on overall plasma equilibrium and confinement. This diagnostic gives a maximum initial potential (soon after injection) of $\lesssim {50}\,\hbox {V}$. This peak negative potential suggests an upper bound on $T_e$, because it is well known that $e\phi \gg kT_e$ in non-neutral plasma. Assuming $e\phi /kT_e \sim 10$, this approximates $T_e$ to be $\sim {5}\,\hbox {eV}$ soon after injection.

To further establish the above temperature estimation, we externally puffed two gases with similar mass namely, molecular hydrogen $\hbox {H}_{2}$ and helium $\hbox {He}$; for each gas neutral pressure was varied from ${3\,\times 10^{-9}}\,\hbox {mbar}$ to ${2\times 10^{-8}}\,\hbox {mbar}$. Launched diocotron mode frequency at ${8}\,\hbox {s}$ (during the long attained quiescent phase) is compared with the above listed cases of $P_H$ and $P_L$, and is shown in Fig.  4 (right). It can be seen that as respective neutral pressure increases, the mode frequency decreases for both $\hbox {H}_{2}$ and $\hbox {He}$, but neither any perceptible instability nor an increase in frequency is observed in both cases. In fact, comparatively we find that for a given pressure, the frequencies are consistently lower in case of $\hbox {H}_{2}$ (red circles) than $\hbox {He}$ (blue square), even though hydrogen has lower ionization potential (${15.6}\,\hbox {eV}$) than Helium (${25.4}\,\hbox {eV}$). The lower frequencies in case of hydrogen can only be explained by transport driven loss of electrons resulting from elastic e-n collisions that dominates over inelastic collisions (resulting in ionization) in the temperature range of interest (1–${100}\,\hbox {eV}$). This is evident from Fig.  4 (left) since the rate coefficient of momentum transfer is more than that of the electron impact ionization for respective gases ($T_e < {40}\,\hbox {eV}$). It can also be seen from Fig.  4 (left), that the total momentum transfer (MT) rate coefficient for $\hbox {H}_{2}$ starts exceeding that of $\hbox {He}$ for $T_e \lesssim {20}\,\hbox {eV}$, and in fact becomes more pronounced as $T_e$ falls below ${10}\,\hbox {eV}$ and approaches ${1}\,\hbox {eV}$. We can therefore safely presume that $T_e$ could lie between 2–${5}\, \hbox {eV}$, thus validating our above estimates from the simple particle balance model and HILP.

**Figure 5:**
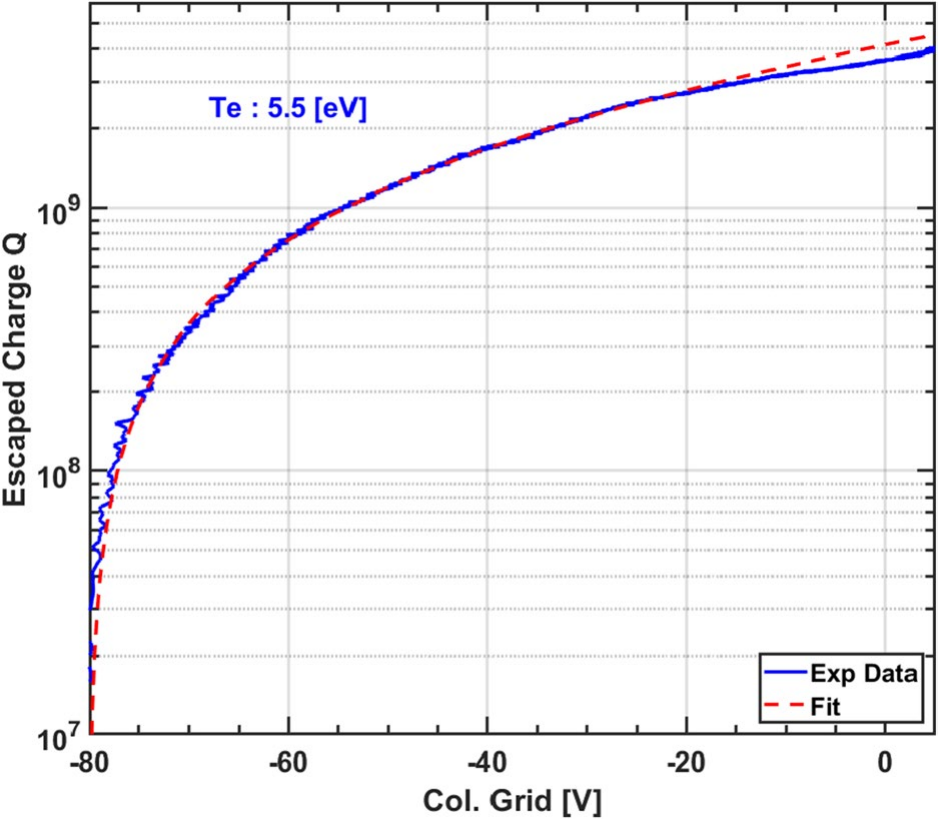
Preliminary estimation: parallel temperature of electron plasma in SMARTEX-C.

Estimation of electron temperature from this model is further validated by preliminary measurement of parallel temperature. This has been done by measuring the number of trapped electrons energetic enough to escape past the confinement potentials of SMARTEX-C. In this commonly called evaporative dump technique^38^, the charge collector^39^ (collector grid + collector shield) voltage is ramped up slowly to ground ($V = 0$ in ${1}\, \upmu \hbox {s}$) and the current is measured due to charges falling on the charge collector. Number of charges that escape are obtained as a function of the potential barrier, by integrating the current signal. If the distribution is assumed Maxwellian, then on a semi-log scale the charge versus voltage plot will be linear and its slope can give us an estimate of parallel temperature of the electron cloud. Such a plot and estimation is shown in Fig.  5. But, there are several underlying assumptions and limitations of the technique as adapted here. First, the plasma is assumed to be in thermal equilibrium with a Maxwellian distribution. Although one may argue about the possibility of a non-Maxwellian distribution, but since the e–e collision frequency is much larger than the total (elastic and inelastic) e–n collision frequency during the bulk of the discharge duration (read quiescent phase), the possibility of creating a substantially non-Maxwellian distribution is unlikely. While such a distribution may arise close to the end-electrodes due to the applied constant negative bias, it will not exist in regions much beyond the sheath thickness. Second, it is assumed that collection of electrons overcoming the bias potentials is based on their energy distribution and not influenced by their proximity to the collector. To address this, the linear machines use separate gating electrode and the collector is placed away at a distance large enough (comparable to the length of the plasma) so that all the electrons travel nearly the same distance. But this has not been possible in SMARTEX-C. Third, being a non-neutral plasma it is highly likely that the electrons will have a larger component of potential energy as compared to their kinetic energy; and when this plasma is dumped on the collector, the potential energy too gets converted into the kinetic energy and thus gives an incorrect estimation of $T_e$. To account for this, the linear devices generally correct the measured temperature by a factor which is determined by a particle-in-cell (PIC) simulation, but no such correction has been done for our measurements. However, despite the above stated limitations we obtain as shown in Fig.  5, a $T_e$ of ${5.5}\,\hbox {eV}$, which may well be regarded as its upper limit. Note that a small reworking of the electron density solution for these temperatures suggests an over-estimation of $\lesssim 25\%$ in the confinement time.

In addition to preliminary measurement of parallel temperature, the charge collector diagnostic can also provide a measurement of the total charge stored in the trap at any instant of time. In our experiments the total stored charge *Q* has been measured in the initial phase and is found to be nearly ${2}\,\hbox {nC}$. One can now get an estimate of the average plasma density as $n_e = Q/(2 \pi ^{2} eR_0r^{2})$, where *e* is the electronic charge. Assuming $R_0$ to be the device major radius of ${13.5}\,\hbox {cm}$ and the initial plasma radius *r* to be maximally the size of the (circular) injector with a radius of ${5}\,\hbox {cm}$ during this period, the total charge then corresponds to $n_e \sim {1.9\,\times 10^{6}}\,\hbox {cm}^{-3}$. With the temperature estimate of $\sim {5.5}\,\hbox {eV}$ given above, the Debye length of the plasma $\lambda _D = \sqrt{\varepsilon _0 k T_e/n_e e^{2}}$ works out to be $\sim {1.3}\,\hbox {cm}$. Although comparison of $\lambda _D$ with *r* may suggest a slightly tenuous plasma at this stage, we believe that $\lambda _D$ would in fact soon evolve to be smaller because (i) due to the well known inward shift, a decrease in $R_0$ would yield a proportionally higher $n_e$, (ii) $T_e$ rapidly drops to $\lesssim {2}\,\hbox {eV}$ without any significant reduction in density as attested by our very long confinement times.

One could also attempt to infer density from peak floating potential of HILP or the diocotron mode frequency from capacitive probes. Besides the use of probes to measure plasma parameters being non-trivial in a pure electron plasma^40^, HILP as configured in our present experiment would overestimate the density, as the potential measurements are carried out during the injection phase. On the other hand, estimation of density from diocotron frequency $f_D$ would require knowing the relation between $f_D$ and $n_e$ for a small aspect ratio torus. In its absence, using the known such relation derived earlier for cylindrical configurations will give an inaccurate density estimate.

## Discussion and conclusion

It must be pointed out that estimation of plasma lifetime and its correction due to possible ionization (through temperature measurements) has not been reported in our previous work^32^, or from contemporary large aspect ratio toroidal experiment LNT-II^29^ (which assumed $T_e$ to be ${1}\,\hbox {eV}$) as well as from the longest reported confinement time results of the levitated dipole experiment RT-1^34^. In our experiments we have thus found that the continuous decrease of frequency with time in the two (pressure) cases can be attributed to both e–e and elastic e–n scatterings, because these collisions eventually lead to cross-field plasma transport through a redistribution of momentum and energy. In the present experiment, we find the possibility of elastic e–n collisional contribution to dominate that from inelastic ones, because despite the $P_H$ case having a higher neutral content it continues to succumb more to the transport and associated particle loss with a continuous decline of frequency rather than become unstable or lead to an increase in frequency. The likelihood of this is corroborated by Fig.  4 (left) which shows that if $T_e$ is low enough, the rate coefficient for elastic e-n scattering dominates impact ionization of $\hbox {H}_{2}$ by more than two orders of magnitude. We believe that the comparatively faster depletion of electrons in $P_H$ case also impacts the space-charge electric field and leads to a lower confinement time, compared to the $P_L$ case.

It is tempting to directly compare the observed confinement time with the theoretical limit based on the MPT theory of Crooks and O’Neil. We have not carried out such a comparison in this paper because the theory provides an analytical estimate of only the radial particle flux that would accrue due to the magnetic pumping mechanism. A robust and reliable measurement of plasma potential and/or density profiles, especially during the quiescent phase would therefore be necessary to calculate the derived flux and offer a correct comparison with the theory. While it is true that our earlier paper presented such a comparison for SMARTEX-C^32^, the confinement time used therein was based on the dimensional analysis of Marler et al.^29^, used for LNT-II^29^. Using the MPT-based flux, the confinement time was evaluated to be $\tau = 0.02 R_0^{2} \sqrt{T_e}$, with $R_0$ being the device major radius (in cm) and $T_e$ in eV. However, upon a closer inspection, we find that the confinement time re-evaluates to $\tau = 2.2 R_0^{2} \sqrt{T_e}/\left( \ln \Lambda + 0.75\right)$ (see Supplementary Appendix for derivation), and gives an order of magnitude higher limit. This results in $\tau \sim {43 - 74}\, \hbox {s}$ for our trap ($-$ $T_{e} = {2}$–${6}\, \hbox {eV}$). Here, it must be mentioned that for evaluating the radial potential gradient $\partial \phi /\partial r$ to be used in the MPT radial flux, the dimensional analysis based estimate ignored the contribution from $\partial ^{2} \phi /\partial r^{2}$, which may not be an accurate approximation as there could be significant contributions due to shear in the (poloidal) $E \times B$ velocity.

In conclusion, recent experiments on SMARTEX-C have led us to confine pure electron plasmas with a purely toroidal magnetic field for an unprecedented time exceeding ${100}\, \hbox {s}$. Lower pressures and low injection energies have ensured substantially reduced ionization. Although, at injection energy of ${100}\, \hbox {V}$ there is some evidence of an additional (small) contribution to the electron population through initial ionization with its tell-tale evidence in the form of an ion-driven instability, it quickly fades out due to rapid cooling by inelastic e–n collisional losses. The plasma then quickly settles into a quiescent state and the confinement time is found to be above ${100}\, \hbox {s}$ even after accounting for about 25$\%$ overestimation (due to uncertainties in temperature measurements). Using a simple particle balance model, the plasma temperature during the long quiescent phase is estimated to be $\lesssim {2}\, \hbox {eV}$. At this stage, the charge evolution and its loss mechanisms is expected to be dictated by e–e and elastic e–n scattering. An analysis based on the conservation of adiabatic invariants, the MPT theory argues that, mediated by e–e collisions, the plasma expands in the radial direction due to Joule heating at the expense of the electrostatic potential energy of the plasma. The radial flux $\Gamma _r$ derived for such a transport depends inversely on the radial electric field, and a confinement time may be deduced for such a plasma from a particle inventory model as $1/r^{2}\Gamma _r$ (*r* is the plasma radius). Although e–e collisions is presumed to be responsible for eventually limiting the lifetime of plasmas in an in-homogeneous magnetic field as suggested by MPT, we believe that it will prevail only at background pressures lower than that in the present experiments. To delineate the transport due to MPT (mediated by e–e collisions) and elastic e–n scattering, an accurate estimate of the outward flux obtained through spatially and temporally resolved plasma density, electric field and temperature measurements will be required.

It would be noteworthy to emphasize the changes in the experimental set-up that have led to this result with respect to our previous result of ${2.14} \pm {0.1}\,\hbox {s}$ confinement time^32^. The old experimental set-up had a vacuum of ${1.5} \pm {0.1\times 10^{-8}}\, \hbox {mbar}$, un-treated surface finish of the vacuum vessel, B-field of 400 Gauss with a droop of ${12}\%$ per second and temporal ripple of ${1} \%$. Following changes have now been made in SMARTEX-C to achieve the reported confinement time. Vacuum vessel and all trap electrodes have been electro-polished to achieve a low outgassing rate. Two NEG pumps have been installed in the main vacuum chamber, as the aim is to preferentially pump light gases like hydrogen, as $\hbox {H}_{2}$ is the most dominant background gas. All of these measures, have resulted in bringing stable operating vacuum of ${4.0} \pm {1.0\times 10^{-10}}\, \hbox {mbar}$. New DC power-supply has a temporal ripple of $< {0.2}\%$ and droop $< {0.1}\%$ for similar magnetic field values. The re-designed trap components have been fabricated with maximum symmetry possible in the mechanical alignment. These efforts should minimize the asymmetry and ripple induced transport in SMARTEX-C. Hence, steady-state droop free magnetic field, improved vacuum scenario and symmetric arrangement of trap components have resulted in the overall improvement in the particle confinement time in the upgraded SMARTEX-C. Future experiments and results from contemporary traps will also help us to compare and may throw some light if unique features of SMARTEX-C, such as, partial torus and tight aspect ratio have any role to play.

### Supplementary Information

Supplementary Information.

## Data Availability

The data-set used and/or analyzed during the current study available from the corresponding author on reasonable request.
