# Supplementary material for: Unprecedented confinement time of electron plasmas with a purely toroidal magnetic field in SMARTEX-C
Source: Sci Rep. 2023 Nov 3;13:19038. doi: 10.1038/s41598-023-44849-2 (PMC10624693; doi:10.1038/s41598-023-44849-2)
Supplement: Supplementary file 1 — Supplementary Information. [file 41598_2023_44849_MOESM1_ESM.pdf]

APPENDIX

The particle confinement time τ is typically derived from a particle balance equation by ignoring sources and sinks, as

$$\frac{\partial n}{\partial t} + \nabla \cdot \Gamma = 0 \tag{1}$$

where n is the (electron) density and Γ is the particle flux. Integrating Eq.(1) over the entire plasma volume V, we get

$$\frac{d}{dt} n_{\rm av} + \frac{\Gamma_r A}{V} = 0 \tag{2}$$

where $n_{\rm av} = \frac{1}{V}\int ndV$ is the volume averaged density, A is the plasma surface area, and Γ_r is the radial particle flux. Substituting $V = \pi r^2(2\pi R_0)$ and $A = 2\pi r(2\pi R_0)$ for a toroidal geometry, where r and R_0 are the plasma and device major radii respectively, we get an estimate of the confinement time, and derived by Marler *et.al*[1] as,

$$\tau = n_{\rm av} 2\pi^2 r^2 R_0/(\Gamma_r 4\pi^2 r R_0) \tag{3}$$

Note that Marler *et.al* have replaced the actual plasma radius r by the device minor radius a and replaced the radial particle flux Γ_r by that derived theoretically by Crooks and O'Neil [2] using the magnetic pumping transport mechanism for a toroidally confined pure electron plasma, i.e.,

$$\Gamma_r = \frac{1}{2}\nu_{\perp,||} n(r) \frac{kT}{-e\partial\phi/\partial r}\left(\frac{r}{R_0}\right)^2 \tag{4}$$

where $\nu_{\perp,||}$ is the equipartition rate, T is plasma temperature (in eV), $\partial\phi/\partial r$ is the potential gradient. Assuming plasma transport and electric field to be predominantly radial in nature, and ignoring spatial gradients in toroidal and poloidal directions, $\partial\phi/\partial r$ can then be readily evaluated from the Poisson equation (in Gaussian units) as,

$$\frac{1}{r}\frac{\partial}{\partial r}\left(r\frac{\partial\phi}{\partial r}\right) = 4\pi e n \tag{5}$$

where e is the electronic charge. Upon ignoring contributions from $\partial^2\phi/\partial r^2$, Marler *et.al* then approximated $\partial\phi/\partial r \sim 4\pi n e a$ for substitution in Eq. (4). Here, it must be noted that the collision rate $\nu_{\perp,||}$ in uncorrelated regimes is well approximated by[3],

$$\nu_{\perp,||} = \frac{8\sqrt{\pi}}{15} n b^2 \bar{v}\left[\ln\left(\sqrt{2}/\bar{\kappa}\right) + 0.75\right] \tag{6}$$

where $b = e^2/kT$ is the classical distance of closest approach, $\bar{v} = \sqrt{kT/m}$, $\bar{\kappa} = \sqrt{2}\,(b/r_c)$, with $r_c = \bar{v}/\Omega_c$ and $\Omega_c = eB/mc$ as the mean cyclotron radius and cyclotron frequency,

respectively. Plugging in the constants, we get $\nu_{\perp,||} = 8.22 \times 10^{-7} (\ln \Lambda + 0.75)\, nT^{-3/2}$, where $\Lambda = 1.656 \times 10^7 T^{3/2}/B$, with n in cm^{-3}, T in eV, and B in Gauss. Substituting back in Eq.(4), we get $\Gamma_r = 0.227\,(\ln \Lambda + 0.75)\, nT^{-1/2} r/R_0^2$. Utilizing the approximation of Marler *et.al* i.e., $\partial\phi/\partial r \sim 4\pi nea$ (with $r \sim a$ and $n_{av} \sim n$), and replacing it all back in Eq.(3), we get the following relation,

$$\tau = \frac{2.2}{\ln \Lambda + 0.75} R_0^2 \sqrt{T} \tag{7}$$

Eq.(7) is approximately one order of magnitude higher than that quoted by Marler *et.al.* Assuming electron temperature T of 2 eV, $R_0 = 13.5$ cm, and $B = 200$ Gauss, for SMARTEX-C, we get $\tau \sim 43$ s.

REFERENCES

[1] J. P. Marler and M. R. Stoneking, "Confinement time exceeding one second for a toroidal electron plasma," Physical Review Letters **100**, 155001 (2008).

[2] S. M. Crooks and T. M. O'Neil, "Transport in a toroidally confined pure electron plasma," Physics of Plasmas **3**, 2533–2537 (1996).

[3] F. Anderegg, D. H. E. Dubin, T. M. O'Neil, and C. F. Driscoll, "Measurement of correlation-enhanced collision rates," Phys. Rev. Lett. **102**, 185001 (2009).
